# Extent of MGMT promoter methylation correlates with outcome in glioblastomas given temozolomide and radiotherapy

**DOI:** 10.1038/sj.bjc.6605127

**Published:** 2009-06-16

**Authors:** J Dunn, A Baborie, F Alam, K Joyce, M Moxham, R Sibson, D Crooks, D Husband, A Shenoy, A Brodbelt, H Wong, T Liloglou, B Haylock, C Walker

**Affiliations:** 1 Department of Surgery and Oncology, School of Cancer Studies, University of Liverpool, Liverpool L3 9TA, UK; 2 Walton Centre for Neurology and Neurosurgery, Lower Lane, Liverpool L9 7LJ, UK; 3 Clatterbridge Centre for Oncology, Clatterbridge Hospital, Bebington, Wirral CH63 4JY, UK

**Keywords:** MGMT, pyrosequencing, methylation, glioblastoma, temozolomide, glioma, survival

## Abstract

**Background:**

Epigenetic silencing of O^6^-methylguanine-DNA-methyltransferase (MGMT) by promoter methylation is associated with improved survival in glioblastomas treated with alkylating agents. In this study, we investigated MGMT promoter methylation in glioblastomas treated with temozolomide and radiotherapy in a single UK treatment centre.

**Methods:**

Quantitative methylation data at individual CpG sites were obtained by pyrosequencing for 109 glioblastomas.

**Results:**

Median overall survival (OS) was 12.4 months with 2-year survival of 17.9%. Pyrosequencing data were reproducible with archival samples yielding data for all glioblastomas. Variation in methylation patterns of discrete CpG sites and intratumoral methylation heterogeneity were observed. A total of 58 out of 109 glioblastomas showed average methylation >non-neoplastic brain in at least one clinical sample; 86% had homogeneous methylation status in multiple samples. Methylation was an independent prognostic factor associated with prolonged progression-free survival (PFS) and OS. Cases with methylation more than 35% had the longest survival (median PFS 19.2; OS 26.2 months, 2-year survival of 59.7%). Significant differences in PFS were seen between those with intermediate or high methylation and unmethylated cases, whereas cases with low, intermediate or high methylation all showed significantly different OS.

**Conclusions:**

These data indicate that MGMT methylation is prognostically significant in glioblastomas given chemoradiotherapy in the routine clinic; furthermore, the extent of methylation may be used to provide additional prognostic stratification.

Glioblastoma accounts for 60–70% of gliomas, but despite advances in therapy these tumours remain associated with poor prognosis (Anderson *et al*, 2008). Until recently the mainstay of treatment was biopsy with cytoreductive surgery where possible, followed by radiotherapy. However, a recent phase III clinical trial (EORTC 26981/22981 & NCIC CE.3) of concurrent temozolomide and radiotherapy followed by adjuvant temozolomide for newly diagnosed glioblastoma patients showed a considerable advance, achieving a median survival of 14.6 months and 2-year survival of 26% (Stupp *et al*, 2005). This treatment was recommended in 2006 in the UK NICE guidelines for good performance status glioblastoma patients, but the impact of chemoradiotherapy on outcome in large cohorts treated in the routine clinic has not been reported.

Alkylating chemotherapeutic agents cause DNA damage by the transfer of alkyl groups at several sites within DNA, including the O^6^ position of guanine. The O^6^-methylguanine-DNA-methyltransferase (MGMT) gene plays a fundamental role in maintaining genomic integrity by encoding for a DNA repair protein that removes alkyl groups from O^6^-guanine (Hegi *et al*, 2008; Verbeek *et al*, 2008). Epigenetic silencing by promoter methylation results in decreased MGMT expression and correlates with improved survival in glioma patients treated with alkylating agents (Esteller *et al*, 2000; Chinot *et al*, 2007; Criniere *et al*, 2007; Eoli *et al*, 2007; Hegi *et al*, 2008). The prognostic significance of MGMT promoter methylation has been shown in two chemoradiotherapy clinical trials; first in a phase II study testing concomitant and adjuvant temozolomide and radiation (Hegi *et al*, 2004) and subsequently in EORTC 26981/22981 & NCIC CE.3 (Hegi *et al*, 2005). In the later study, MGMT promoter methylation was an independent favourable prognostic factor and patients whose tumour contained a methylated MGMT promoter had median survival of 21.7 months and 2-year survival of 46%, when treated with temozolomide and radiotherapy. These studies suggest that determination of MGMT methylation status maybe an important factor in determining which glioblastoma patients should receive chemoradiotherapy (Gorlia *et al*, 2008), but its prognostic significance in the routine clinical setting is not clearly established.

Methylation-specific PCR (MSP) is widely used to test MGMT promoter methylation; however, in EORTC 26981/22981 & NCIC CE.3 Hegi *et al* (Hegi *et al*, 2005) only achieved methylation data for 67% of samples analysed, representing 36% of cases. The MSP assay is prone to lack of reliability attributed to poor quality archival tissue and small sample size and optimum results are usually obtained with cryopreserved tissues (Hegi *et al*, 2005; Yip *et al*, 2008). A number of improvements (Cankovic *et al*, 2007) and alternative methods including semi-quantative methods have been developed (Jeuken *et al*, 2007; Lorente *et al*, 2008; Vlassenbroeck *et al*, 2008). Pyrosequencing allows highly reproducible quantification of methylation at each CpG site within the chosen amplicon and has been found to be robust when applied to archival samples including glioblastomas (Mikeska *et al*, 2007), but use of pyrosequencing in analysis of extensive glioblastoma cohorts and comparison with outcome data has not been reported.

The aim of this study was to determine whether pyrosequencing may be used to determine MGMT promoter methylation status using archival tissue samples from glioblastomas and to investigate the prognostic significance of MGMT promoter methylation in patients treated in the routine clinic with concurrent temozolomide and radiotherapy followed by adjuvant temozolomide.

## Patients and methods

### Case selection

The study included 109 newly diagnosed, previously untreated glioblastomas WHO grade IV diagnosed between June 2004 and October 2007 (Table 1). These patients had cytoreductive surgery where possible or biopsy at the Walton Centre for Neurology and Neurosurgery before radical treatment at Clatterbridge Center for Oncology with radiotherapy and concurrent temozolomide (75 mg m^−2^ per day) and radiotherapy followed 4 weeks later by adjuvant temozolomide (150 mg m^−2^ for 5 days and increased to 200 mg m^−2^ for 5 days in subsequent cycles depending on blood counts and tolerability, six cycles planned). A total of 60 and 55 Gy were prescribed to 104 and 5 patients, respectively, in 30 fractions. In the preliminary experiments, 12 archival glioblastomas and high-grade glioma cell lines U373, HS683, T98G and U87MG were investigated to validate the pyrosequencing assay. Six non-neoplastic brain samples: two whole brain DNA extracts (AMS Bio D1234035-50, Abingdon, UK) and four temporal lobectomies from epilepsy surgery (two formalin-fixed paraffin-embedded (FFPE); two snap frozen) were also investigated. The study had the ethics committee approval.

### Clinical data

Clinical data were collated retrospectively for all patients treated by chemoradiotherapy within the study period (*n*=115). Response was monitored with computed tomography or magnetic resonance imaging scans after radiotherapy, before the fourth cycle and after the sixth cycle. Scans were performed if there was clinical suspicion of disease progression and at regular 6-monthly intervals during follow-up. PFS and OS were calculated from the date of diagnosis.

### Pathology and tissues

For tumour tissues, a consultant neuropathologist reconfirmed a diagnosis of glioblastoma WHO grade IV and selected suitable samples for analysis by visual microscopic assessment with >70% neoplastic cells and <50% necrosis from intraoperative cytology smear preparations or FFPE blocks for each case. Haematoxylin and eosin or methylene blue stained smears were used as described earlier (Walker *et al*, 2001) scraping the tissue into DNA extraction buffer. Snap frozen tissue was used where available. We aimed to analyse more than one tissue sample for each case, preferably selecting samples from different blocks and/or with different fixation.

### DNA extraction and bisulphite treatment

The DNA was extracted using the Dneasy Blood and Tissue kit (Qiagen cat 69506, Crawley, UK) and quantified by spectrophotometry using a NanoDrop (NanoDrop ND-1000, Thermofisher Scientific, Loughborough, UK). The DNA yields for frozen, smear and FFPE samples were 6.9±7.0 *μ*g, 3.2±4.1 *μ*g and 20.9±22.2 *μ*g, respectively. Bisulphite modification of 1 *μ*g DNA was performed using the EZ DNA methylation kit (Zymo, Orange, CA, USA, D5002). Each bisulphite modification experiment included universal methylated DNA (CpGenome Universal Methylated DNA S7821, Millipore, Watford, UK) as positive control and normal brain DNA as negative control.

### Analysis of MGMT promoter methylation

The pyrosequencing assay was performed as described earlier (Shaw *et al*, 2006). The primers used for amplification of bisulphite-treated DNA were forward: 5′-gGGATAGTTGGGATAGTT-3′ (the first g avoids formation of hairpin loops) and reverse: 5′-biotin-ATTTGGTGAGTGTTTGGG-3′ giving a 99-bp amplicon at genomic position 131 155 467–131 155 565. The PCR analysis was performed in duplicate in 25 *μ*l reaction volume, containing 300 pmol each forward and reverse primer, 2 *μ*l 10 × buffer, 160 *μ*M dNTPs, 0.5 U HotStar Taq polymerase (Qiagen) and 1–2 *μ*l bisulphite-treated DNA. The PCR conditions were as follows: 95°C-15 min; 40 cycles of 94°C-30 s, 50°C-45 s, 72°C-30 s; 72°C-10 min (Dyad, GRI, Braintree, UK). To confirm the correct product before pyrosequencing, 3 *μ*l of PCR products were analysed on a 2% agarose gel, the remaining 22 *μ*l was subjected to pyrosequencing on a PSQ96MA System (Biotage, Uppsala, Sweden) using the primer 5′-GGATATGTTGGGATAGT-3′and PyroGold reagents (Biotage). The Pyro Q-CpG software 1.0.9 (Biotage) was used to analyse data.

Pyrosequencing yields data for 12 CpG sites within the MGMT promoter. For data analysis, the percentage methylation obtained for each CpG was averaged across the 12 CpGs in duplicate PCR reactions (average methylation per sample). For comparisons with clinical data, glioblastomas were considered methylated if they had at least one sample with average methylation ⩾9% (⩾mean±2 s.d. for non-neoplastic brain) in more than one independent bisulphite modification. Unmethylated cases had average methylation <9% in all samples. The average methylation per case was calculated by averaging the average methylation per sample for methylated samples for that case. Methylation-specific PCR assays were carried out as described by Hegi *et al* (2005).

### Statistics

Statistical analysis was performed using SPSS (Chicago, IL, USA). Unsupervised hierarchical cluster analysis was performed in Gene Spring, using Euclidean distance and centroid linkage. Survival data were calculated from the date of diagnosis. Kaplan–Meier survival curves were obtained and differences in PFS or OS were tested for statistical significance using the log-rank test. Univariate Cox regression analysis was used to determine whether MGMT methylation status, gender, age, extent of surgery and performance status had prognostic significance with respect to outcome. Cox regression multivariate analysis for factors significantly associated with survival in univariate analysis was by forward stepwise entry of parameters at a significance of 0.05 for entry and 0.01 for removal. Two-tailed *P*-values are quoted. Methylated cases were dichotomised using receiver operator characteristic (ROC) plots comparing average methylation per case with the Cox regression survival function for OS.

## Results

### MGMT methylation analysis

The pyrosequencing assay for MGMT methylation was initially validated in a series of dilutions of *in vitro* methylated: unmethylated DNA. The reproducibility of the assay, measured by multiple repetitions at various dilutions was calculated as 3%. The sensitivity of the assay, defined as the maximum tolerable dilution within the reproducibility range was 5%. The next validation phase included cell lines of known MGMT methylation status: U373, T98G, U87MG and HS683 showed methylation averaged across the 12 CpGs analysed of 42, 42, 64 and 5%, respectively, consistent with their methylation status determined by MSP (data not given). As DNA methylation is tissue specific, we determined the background methylation in two non-neoplastic brain extracts, which showed average methylation of <5%. We then investigated MGMT promoter methylation by pyrosequencing in five glioblastomas with frozen tissue and seven cases each with frozen tissue, intraoperative smears and FFPE samples meeting the selection criteria of >70% neoplastic component and <50% necrosis. Pyrosequencing data were obtained for all classes of sample (Supplementary Figure 1). Six glioblastomas had average methylation <5%, whereas the remainder had methylation levels between 9–49%. A total of 5 out of 7 cases with multiple samples per case showed agreement in methylation between tissue samples. Four non-neoplastic brain and 109 cases treated with temozolomide and radiotherapy were then included in the study. Due to the potential methylation heterogeneity, we aimed to analyse 2–3 separate clinical samples for each case, depending on availability of tissue meeting the selection criteria. Pyrosequencing data were obtained for 264 out of 287 tumour samples from 121 cases, but was unobtainable for 13 out of 123 paraffin-embedded samples and 6 out of 116 intraoperative smears. In 17 cases diagnosed by serial stereotactic biopsy with samples <1 mm^3^, pyrosequencing data were obtained for 5 out of 9 frozen biopsies and 29 out of 31 intraoperative smears. Failures may be because of small sample size with low DNA yield or in FFPE to poor DNA integrity. Despite some sample failure, methylation data were obtained for at least one sample of all clinical cases investigated.

Pyrosequencing data were reproducible, showing good correlation between duplicate PCR reactions from the same bisulphite modification and between two independent bisulphite modifications of the same DNA extract (Supplementary Figure 2). Non-neoplastic brain samples had mean average methylation of 3.2±2.89 s.d. (Supplementary Figure 3), but in order to guarantee a high specificity we employed a conservative approach of defining hypermethylation at ⩾95% reference range (mean normal brain±2 s.d.). A total of 62 out of 121 (51.2%) glioblastomas had average methylation across all CpGs in at least one clinical sample greater than that of non-neoplastic brain (⩾9%) and were classified as methylated. Average methylation in methylated and unmethylated cases was 29±15.5 and 2.9±2.0, respectively. In 22 out of 25 frozen glioblastoma samples, the methylation status obtained by pyrosequencing agreed with that determined by MSP; two were unmethylated by pyrosequencing but showed methylation by MSP and one had 20% methylation but was negative by MSP. In glioblastomas, there was considerable variation in the methylation of individual CpG sites within amplicons and between cases (Supplementary Figure 4a and b), with CpG11 showing the highest methylation and CpG7 the least. To determine whether glioblastomas have characteristic patterns of CpG methylation, methylation at individual CpGs was normalised to the average methylation for that sample and unsupervised hierarchical analysis performed, but discrete clusters were not obtained (data not given).

### MGMT methylation heterogeneity

**Figure 1:**
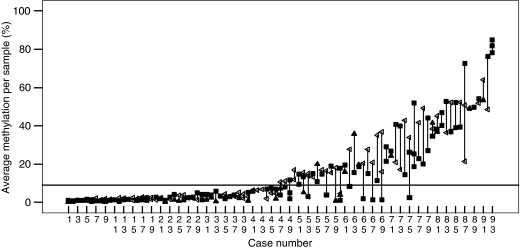
Intratumoral methylation heterogeneity seen in cases with analysis of multiple samples per case. All samples in cases 1–46 had methylation <9% and were classified unmethylated; whereas cases 47–93 showed methylation ⩾9% in at least one tissue sample per case and were considered methylated. ▴ snap frozen tumour tissue; ◃ FFPE samples; ▪ intraoperative cytology smear preparations. The reference line at 9% is the cut-off used to distinguish methylated from unmethylated cases.

Inter and intratumoral heterogeneity in the extent of MGMT methylation was observed. Data were obtained for multiple tissue samples in 93 glioblastomas; some had marked variation in methylation between separate tissue samples, whereas others showed close agreement (Figure 1). Where intratumoral heterogeneity was observed, the analysis was repeated and an additional tissue sample was selected for analysis where available. For FFPE and frozen tissue, the histology was reassessed to ensure that the selection criteria had been met. No obvious morphological differences were evident to account for MGMT methylation heterogeneity. Eighty (86%) had the same methylation status in all samples analysed. A total of 13 cases showed methylation >9% in at least one, but not all clinical samples; in 11 out of 12 methylation was seen in FFPE samples with intraoperative smears or frozen tissue samples being unmethylated; in 9 out of 13 the methylated samples had methylation <21%. In one case with distinct astrocytic and oligodendroglial histology both components were methylated with 20.5 and 21.5%, respectively.

### MGMT methylation and clinical outcome in glioblastomas treated with chemoradiation

**Figure 2:**
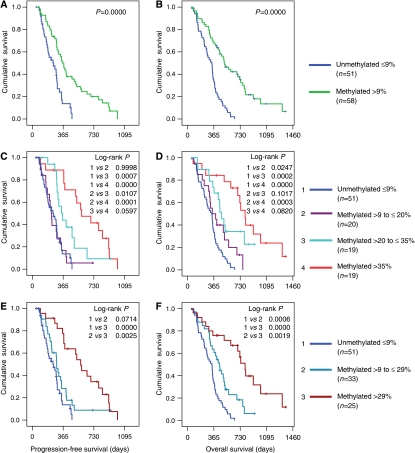
Comparison of MGMT methylation with (**A**, **C** and **E**) progression-free survival and (**B**, **D** and **F**) overall survival. In (**A** and **B**) methylated cases are compared with unmethylated cases. In (**C** and **D**) methylated cases were ordered according to degree of methylation and divided into three groups. In (**E** and **F**) methylated cases were divided into two groups according to a cut-off determined by ROC analysis.

Of 115 patients treated with chemoradiation during the study period, 15 (13%) had temozolomide stopped during radiotherapy, due to clinical deterioration (2) or toxic effects (9) [missing data (4)]. 83 (72%) patients received adjuvant temozolomide (median 4 cycles, range 1-6) with 41 (50%) completing 6 cycles. Adjuvant temozolomide was stopped early due to disease progression (21), clinical deterioration (9), death (2), toxic effects (6) or other (4). Four patients had histology elsewhere and two with inadequate tissue were not included. Methylation data were obtained for 109 cases of which 58 (53.2%) were methylated. Median OS for the complete cohort of 115 patients was 12.8 months, comparing well with the subset with methylation data (Table 1). There was no association between MGMT methylation and gender, age, biopsy *vs* resection or tumour location. The MGMT promoter methylation was highly significantly associated with prolonged PFS and OS (Figure 2A and B; Table 2). Surgery, but not age or performance status influenced outcome. Resected cases had prolonged OS but not PFS compared to those with biopsy only (Log-rank PFS: *P*=0.312; OS: *P*=0.0002). The MGMT status was an independent prognostic variable for both PFS and OS (Cox Regression hazard ratio (methylated cases relative to unmethylated cases): PFS - 0.37 (95% CI 0.23-0.61), *P*=0.0001; OS - 0.25 (95% CI 0.15-0.40), *P*=0.0000).

### Extent of methylation and clinical outcome

In order to determine whether the extent of methylation may be used to provide further prognostic stratification, the average methylation per case was compared with clinical outcome. When methylated cases were ranked according to methylation and divided into three groups, all groups had a longer OS than did the unmethylated cases, and those with the highest methylation (>35%) showed the longest PFS and OS (median 26.2 months) with a 2-year survival of 59.7% (Figure 2C and D, Table 2), suggesting that the extent of methylation impacts on survival. Receiver operator characteristic analysis used to separate methylated cases into two prognostic groups yielded a cut-off of 29.4% with 71.4% sensitivity and 63% specificity (ROC area 0.759 *P*=0.001). Kaplan–Meier plots for PFS and OS using this cut-off are shown in Figure 2E and F. Log-rank tests showed highly significant differences in OS between unmethylated and both methylated groups and between cases with low *vs* high methylation. The 2-year survival of the highly methylated group was 56.7% with median OS at 26.2 months (Table 2). Progression-free survival was highly significantly different between low and high methylated groups and between highly methylated and unmethylated cases. In the analysis above, the percentage methylation was averaged between methylated tissue samples when methylation data were available for more than one clinical sample per case. Similar Kaplan–Meier plots were obtained and conclusions supported when the percentage methylation for the sample with the highest methylation for each case was used for comparison with outcome data (data not given). As methylation varied across CpG sites and between cases, unsupervised hierarchical cluster analysis was performed to identify groups of tumours with similar methylation for comparison with outcome data (Supplementary Figure 4b and c). Three main clusters were identified: cluster 1 corresponded to tumours classified as unmethylated; cluster 2 had cases with lower levels of methylation; cluster 3 had cases with high methylation seen in the majority of CpG sites. Survival curves were similar to that seen for groups in Figure 2E and F, but the highest methylated cluster had a median survival of 23.8 months and a 2-year survival of 49.5%.

## Discussion

Although numerous studies have reported associations between MGMT and survival in glioblastomas treated with alkylating agents, few reports support the findings of EORTC 26981/22981 & NCIC CE.3 in large cohorts treated with temozolomide and radiotherapy (Hegi *et al*, 2005; Stupp *et al*, 2005; Brandes *et al*, 2008). Given the importance to the clinical management of glioblastoma patients, experience in the routine clinic is essential for these advances to have full clinical benefit. Our cohort represents consecutive patients treated in a single centre over a 40-month period except six patients and comparison of survival data supports little selection bias in the study. The cohort with methylation data had similar median age and range, performance status and proportion of patients with biopsy *vs* debulking surgery compared with Stupp *et al* (2005). Progression-free survival was 9.7 months compared with 6.9 months reported by Stupp *et al* (Stupp *et al*, 2005), which may reflect response evaluation and follow-up achieved in routine practice. Overall survival was 12.4 months in contrast to 14.6 months, but this is not an unexpected finding as outcome in clinical trials is often somewhat better than that obtained in a routine clinical environment.

MGMT levels in tumours have been measured by the assessment of protein and gene expression, analysis of enzyme activity or investigation of epigenetic silencing through promoter methylation. However, most studies report poor agreement between these methods (Brell *et al*, 2005; Maxwell *et al*, 2006; Preusser *et al*, 2008; Rodriguez *et al*, 2008; Sasai *et al*, 2008; Yachi *et al*, 2008) and most correlations with outcome have been obtained through investigation of promoter methylation (Hegi *et al*, 2008; Preusser *et al*, 2008). Unlike the MSP assay used in the majority of clinical studies, pyrosequencing allows highly reproducible quantitative evaluation of methylation at discrete CpG sites thereby providing more information on promoter methylation status and facilitating analysis of specific methylation patterns. Analysis is fast and cost effective and, unlike conventional bisulphite sequencing (Grasbon-Frodl *et al*, 2007; Mikeska *et al*, 2007; Parkinson *et al*, 2008), is practical in a diagnostic environment. In our assay, the PCR primers are methylation independent and as little as 5% methylated DNA in a mixture of methylated and unmethylated DNA may be detected. Furthermore, the assay contains an internal control to verify successful bisulphite treatment of the starting DNA. In our study, the criteria for sample selection of >70% neoplastic cells and of <50% necrosis was met through analysis of small samples after macrodissection where necessary or use of serial stereotactic biopsies. Pyrosequencing was successfully used to analyse frozen, paraffin-embedded formalin-fixed and ethanol fixed (intraoperative smear) tissue from our diagnostic archives, including samples of <1 mm^3^ from serial stereotactic biopsies. Data were obtained for at least one sample for all glioblastomas in the clinical series.

The various qualitative and quantitative methods for the analysis of MGMT promoter methylation in clinical samples have been reviewed recently (Yip *et al*, 2008; Preusser, 2009). Mikeska *et al* (Mikeska *et al*, 2007) used a similar pyrosequencing assay but MGMT methylation was analysed at only four CpG sites (CpG 9, 10, 11 and 12 in our assay). They also tested COBRA (combined bisulphite restriction analysis) and SIRPH (SNuPE ion pair-reverse phase high-performance liquid chromatography), but found pyrosequencing the most sensitive, robust and easy to use. The region of the MGMT promoter sequenced in our assay overlaps with that analysed in the MSP assay used by Hegi *et al* (2005), which relies on annealing of primers across nine CpG sites of which the five CpGs in the forward primer correspond to CpGs 5–9 in our assay. Agreement in methylation status between MSP and pyrosequencing was obtained in 88% frozen glioblastoma samples and 100% cell lines tested. Methylation seen in MSP but not in pyrosequencing may be because of the increased sensitivity of MSP detecting methylation in minor populations of tumour cells or false positives, whereas methylation obtained by pyrosequencing and not MSP may reflect the primer positions. More recently a quantitative real-time MSP assay, in which the copy number of methylated MGMT alleles is calculated, improved upon the gel-based MSP assay in terms of reproducibility and use with archival samples, but does not provide methylation data at individual CpG sites (Vlassenbroeck *et al*, 2008).

The MSP assay yields binary data without a quantitative measure of the contribution from unmethylated DNA, which arises from non-neoplastic cells within the tissue or from tumour cells lacking MGMT methylation. Consequently, tumours with high proportions of methylated tumour cells will score the same in the MSP assay as tumours bearing only a few methylated cells, thus underestimating methylation heterogeneity. Pyrosequencing provides quantitative data for the proportions of methylated *vs* unmethylated cytosines at each CpG assayed. As in other studies, we observed heterogeneity in methylation of discrete CpG sites within the amplicon and between different cases (Mikeska *et al*, 2007; Yachi *et al*, 2008). Although multiple samples from the same case showed similar patterns of methylation across the 12 CpGs analysed, many cases showed some variation in the extent of their methylation, indicating a degree of intratumoral methylation heterogeneity in many glioblastomas in our series. As tumour samples were chosen to have >70% neoplastic cells, these data suggest variation in the numbers of methylated tumour cells in different regions of glioblastoma tissues. Similarly, glioblastomas show heterogeneous patterns of MGMT protein expression often with regions within the same tumour displaying widely different staining patterns (Juillerat-Jeanneret *et al*, 2008; Sasai *et al*, 2008). On the basis of the MSP assay and bisulphite sequencing some studies have claimed homogeneity in MGMT status within glioblastomas (Grasbon-Frodl *et al*, 2007), whereas others have reported a degree of intratumoral heterogeneity (Juillerat-Jeanneret *et al*, 2008; Parkinson *et al*, 2008). For example, Parkinson *et al* (Parkinson *et al*, 2008)observed heterogeneity in MGMT promoter methylation in 2 out of 7 glioblastomas sampling from different regions of large glioblastomas and Juillerat-Jeanneret observed heterogeneity in samples taken at least 3 mm apart.

Comparison with clinical outcome was performed in a number of ways. As there was no evidence of discrete methylation patterns across individual CpGs, in the initial analysis cases were considered methylated if methylation averaged across all 12 CpGs in at least one sample was greater than that of non-neoplastic brain. In support of data from EORTC 26981/22981 & NCIC CE.3 and other series treated with temozolomide and radiotherapy (Friedman *et al*, 1998; Hegi *et al*, 2005; Brandes *et al*, 2008), in this cohort methylated cases had prolonged PFS and OS and MGMT status was an independent prognostic factor. However, median OS was 16.8 months with 2-year survival of 35.2%, compared with 21.7 months and 46% in EORTC 26981/22981 & NCIC CE.3 and 43.6 months in the study reported by Brandes *et al* (Brandes *et al*, 2008). These differences may be related to differences in MGMT analysis and sensitivity of the assays. Proportions of MGMT positive cases were 53.2, 45 and 35% in this study and studies reported by Hegi *et al* (Hegi *et al*, 2005)and Brandes *et al* (Brandes *et al*, 2008), respectively. Alternatively, the shorter survival in our study may reflect the routine clinical environment where all patients with appropriate performance status were treated with temozolomide and radiotherapy. Further prognostic stratification was achieved when the extent of methylation (averaged across CpG sites per sample and across methylated samples per case) was considered. This was most readily showed when methylated cases were split into three groups according to extent of methylation. Cases with the greatest methylation had the longest survival. Significant differences in PFS were seen between those with intermediate or high methylation and unmethylated cases, whereas cases with low, intermediate or high methylation all had significantly different OS. Thus, the extent of methylation within the tumour impacts on associations with survival. This may be a reflection of the proportions of cells with silenced MGMT, but the relationship between methylation and expression of active enzyme has been questioned (Maxwell *et al*, 2006; Sasai *et al*, 2008). Alternatively, high numbers of methylated cells may be a marker of less aggressive biology, possibly in association with methylation of other gene promoters (Iafrate and Louis, 2008). Discrimination of methylated cases into two prognostic groups further illustrated this effect. By either method, the group with the highest methylation had median OS at 26.2 months and 2-year survival of 57–60%. In order to take methylation at individual CpG sites into account, unsupervised hierarchical cluster analysis was performed. Unmethylated cases formed one cluster and methylated tumours were divided into two clusters, all of which had significantly different PFS and OS, further supporting the association between extent of methylation and outcome. To our knowledge, this is the first study to associate the extent of MGMT promoter methylation with outcome.

In our study, a cut-off of 9% discriminated outcome between methylated and unmethylated tumours, and a cut-off of 29% could be used to dichotomise methylated cases into two prognostic groups; for clinical use these values require validation in larger series. Although consideration of the extent of methylation allows greater prognostic stratification, a single cut-off in methylation derived from a quantitative assay may be more useful in the clinic. Wiewrodt *et al* (Wiewrodt *et al*, 2008) showed that patients expressing ⩽30 fmol mg^−1^ MGMT protein in the pre-treatment tumour volume had a significantly better response to alkylating therapy than those with MGMT protein above this level. Others have shown that patients with low MGMT protein expression had significantly improved survival compared with those with high expression (Brell *et al*, 2005; Chinot *et al*, 2007; Nagane *et al*, 2007). Vlassenbroeck *et al* (Vlassenbroeck *et al*, 2008) used a real-time MSP assay to determine a clinically relevant cut-off for stratification of glioblastomas into two distinct populations, with prognostic significance in recurrent anaplastic astrocytoma but not glioblastomas treated with temozolomide (Sadones *et al*, 2009).

In this study, we show that analysis of MGMT promoter methylation by pyrosequencing is robust and reliable when used with diagnostic samples and may be used to distinguish two or more prognostic groups in response to chemoradiotherapy. The intratumoral hetereogeneity displayed by many cases illustrates the necessity for careful selection of representative tissue before assay and for analysis of more than one tissue sample per case to exclude false negatives. In our study serial stereotactic biopsy, which delivers multiple samples from a trajectory calculated to traverse the most aggressive parts of the tumour based on MR imaging, and intraoperative diagnosis for both biopsies and resected cases ensured that solid tumour tissue rather than infiltrative edge was available for diagnosis. Where surgical practice differs, samples for analysis with >70% neoplastic cells may be difficult to achieve, but the assay sensitivity would enable detection of methylation in samples with lower tumour content. These data support the potential use of the MGMT pyrosequencing assay for diagnostic purposes, but further inter-laboratory investigation of the criteria for sample selection, extent of heterogeneity and clinically relevant cut-offs in larger series would be essential.

In summary, in this study we have reported outcome data for consecutive glioblastomas patients treated with temozolomide and radiotherapy in a routine UK clinic and have shown that pyrosequencing is a robust and reliable assay for the determination of MGMT promoter methylation using diagnostic archival samples. Patients with methylated tumours had prolonged progression-free and overall survival and the extent of methylation could be used to achieve further prognostic stratification.

## Figures and Tables

**Table 1:** Clinical characteristics of the cohort treated with chemoradiotherapy with methylation data (*n*=109)

**Clinical series**	
*Age*	
Median (range) years	55 (18–68)
	
*Sex*	
Male	72
Female	37
	
*Surgery*	
Biopsy (open : serial stereotactic)	26 (9 : 17) (24%)
Resection	83 (76%)
	
*Performance status (WHO)*	
0	37 (34%)
1	54 (50%)
2	16 (15%)
3	2 (2%)
Alive : dead at study	15 : 94
Progression-free survival^a^ (months)	Median 9.7 months (95% CI 8.8–10.5)
Overall survival^a^ (months)	Median 12.4 months (95% CI 10.7–14.1)
2-Year survival (%)	17.9

a Calculated from date of diagnosis.

**Table 2:** MGMT promoter methylation and outcome

	**Progression-free survival^a^**	**Overall survival^a^**	
	**Median (95% CI) (months)**	**Median (95% CI) (months)**	**2-year survival (%)**
^§^Unmethylated (*n*=51)	8.3 (5.1–11.5)	11.1 (8.8–13.3)	0
Methylated (⩾9% methylation) (*n*=58)	11.8 (9.9–13.6)	16.8 (13.2–20.4)	35.2
			
^#^Methylated (>9 to ⩽20% methylation) (*n*=20)	7.5 (6.3–8.7)	11.3 (6.2–16.3)	13.3
Methylated (>20 to ⩽35% methylation) (*n*=19)	11.8 (8.9–14.6)	15.5 (12.9–18.2)	34.2
Methylated (>35% methylation) (*n*=19)	19.2 (11.9–26.5)	26.2 (23.0–29.5)	59.7
			
^$^Methylated (>9 to <29% methylation) (*n*=33)	9.5 (8.6–10.4)	14.6 (12.0–17.2)	18.4
Methylated (>29% methylation) (*n*=25)	18.8 (15.5–22.2)	26.2 (22.9–29.6)	56.7

a Calculated from date of diagnosis. Kaplan–Meier plots in Figure 2 ^§^a&b; ^#^c&d; ^$^e&f.
