# Supplementary material for: Extent of MGMT promoter methylation correlates with outcome in glioblastomas given temozolomide and radiotherapy
Source: Br J Cancer. 2009 Jun 16;101(1):124–31. doi: 10.1038/sj.bjc.6605127 (PMC2713697; doi:10.1038/sj.bjc.6605127)
Supplement: Supplementary Figures 1–4 [file 6605127x1.pdf]

**Figure 1**

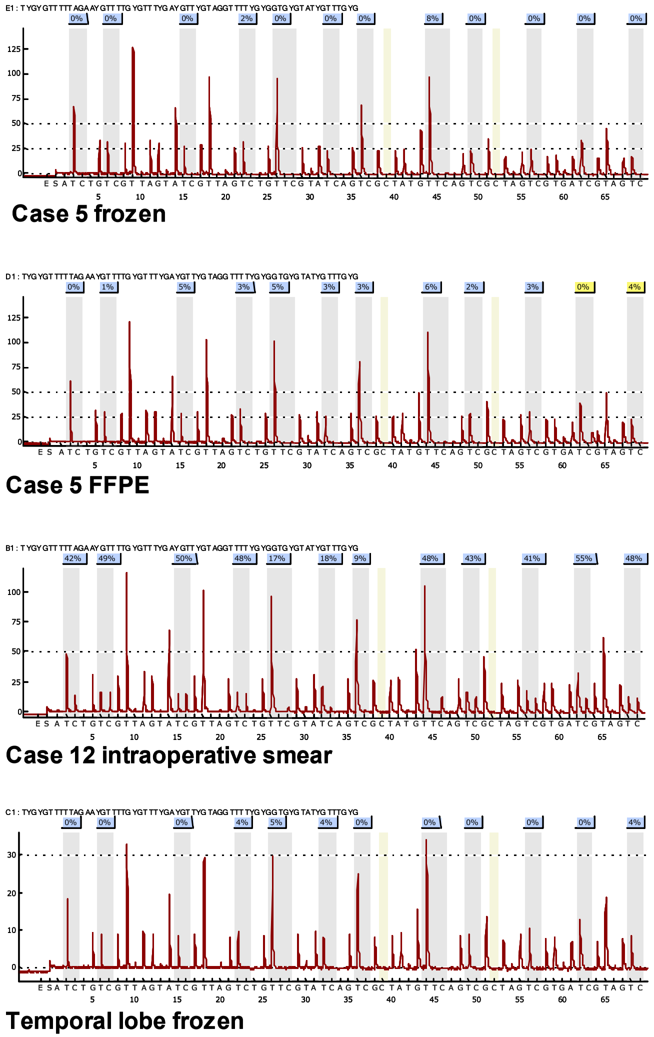


**Figure 1**. Pyrograms representative of all classes of sample: snap frozen (Case 5 and Temporal lobe), formalin-fixed paraffin embedded (Case 5) and ethanol-fixed smear (Case 12) tissues. Percentage methylation at each of 12 CpG sites (marked by grey columns) is given in boxes, which when averaged across each case shows Case 5 and temporal lobe are unmethylated (<9% methylation) and Case 12 is methylated (≥9% methylation). In each pyrogram two yellow shaded columns show the position of internal bisulphite controls.

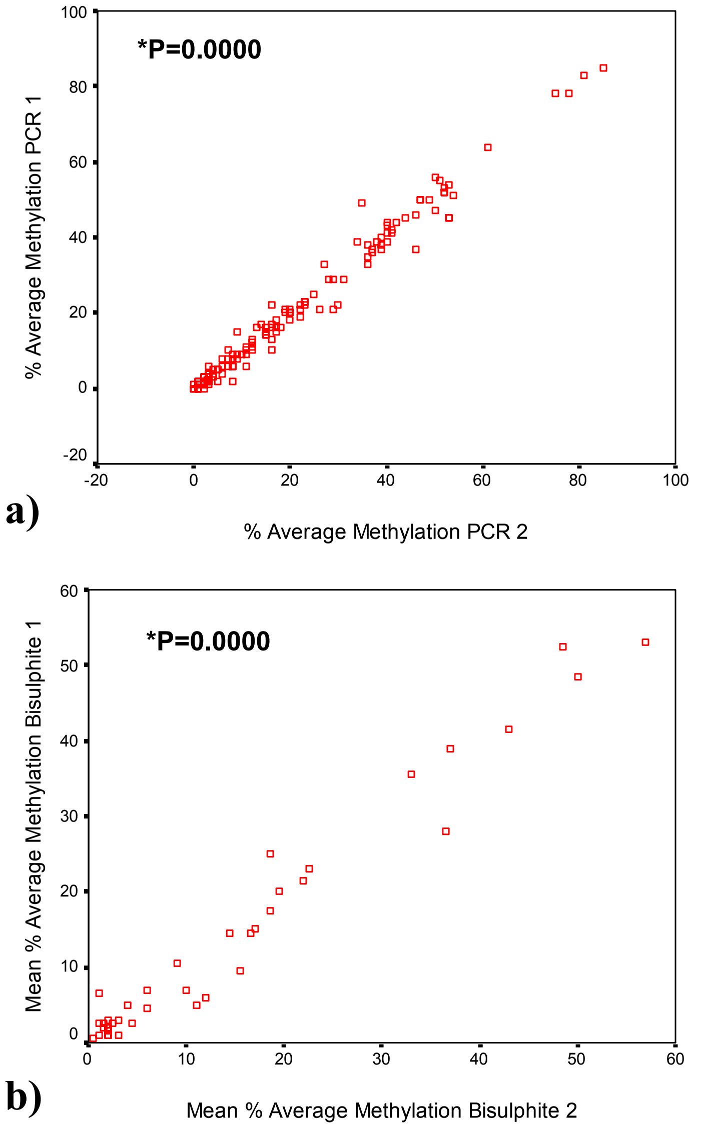


**Figure 2**. Percentage methylation averaged over 12 CpG islands in **a)** 2 independent PCR reactions from the same bisulphite modification and **b)** duplicate PCR reactions from 2 independent bisulphite modifications of the same clinical samples. *Spearman Correlation two-tailed P values.

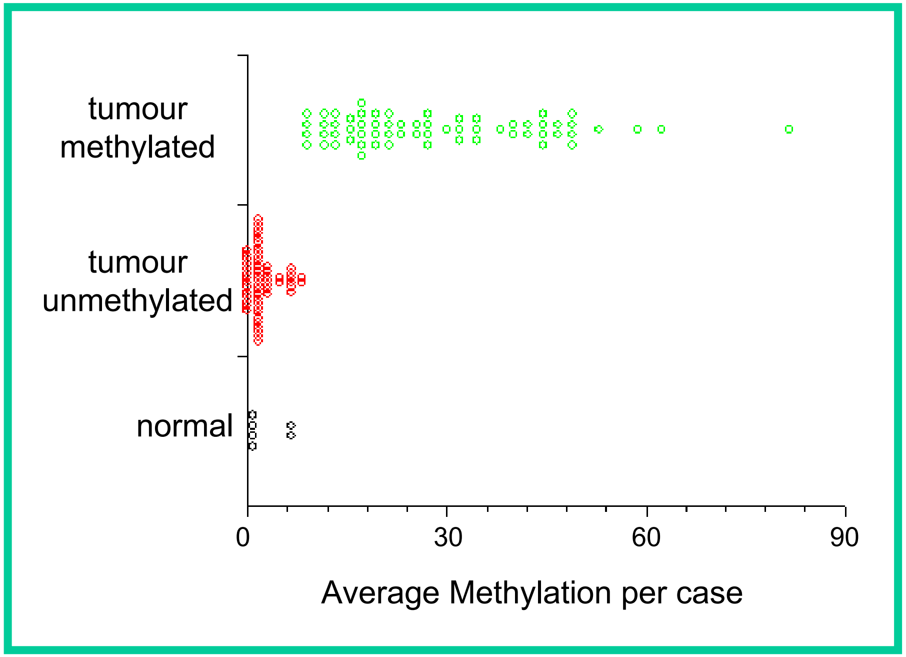


**Figure 3.** Illustration of the distribution of methylation in non-neoplastic brain samples, in unmethylated tumours (<9% methylation) and in tumours considered to methylated with ≥9% methylation. In this analysis the average methylation per case was obtained by averaging the percentage methylation seen in 1-3 methylated tissue samples for each case where data for multiple samples was available.

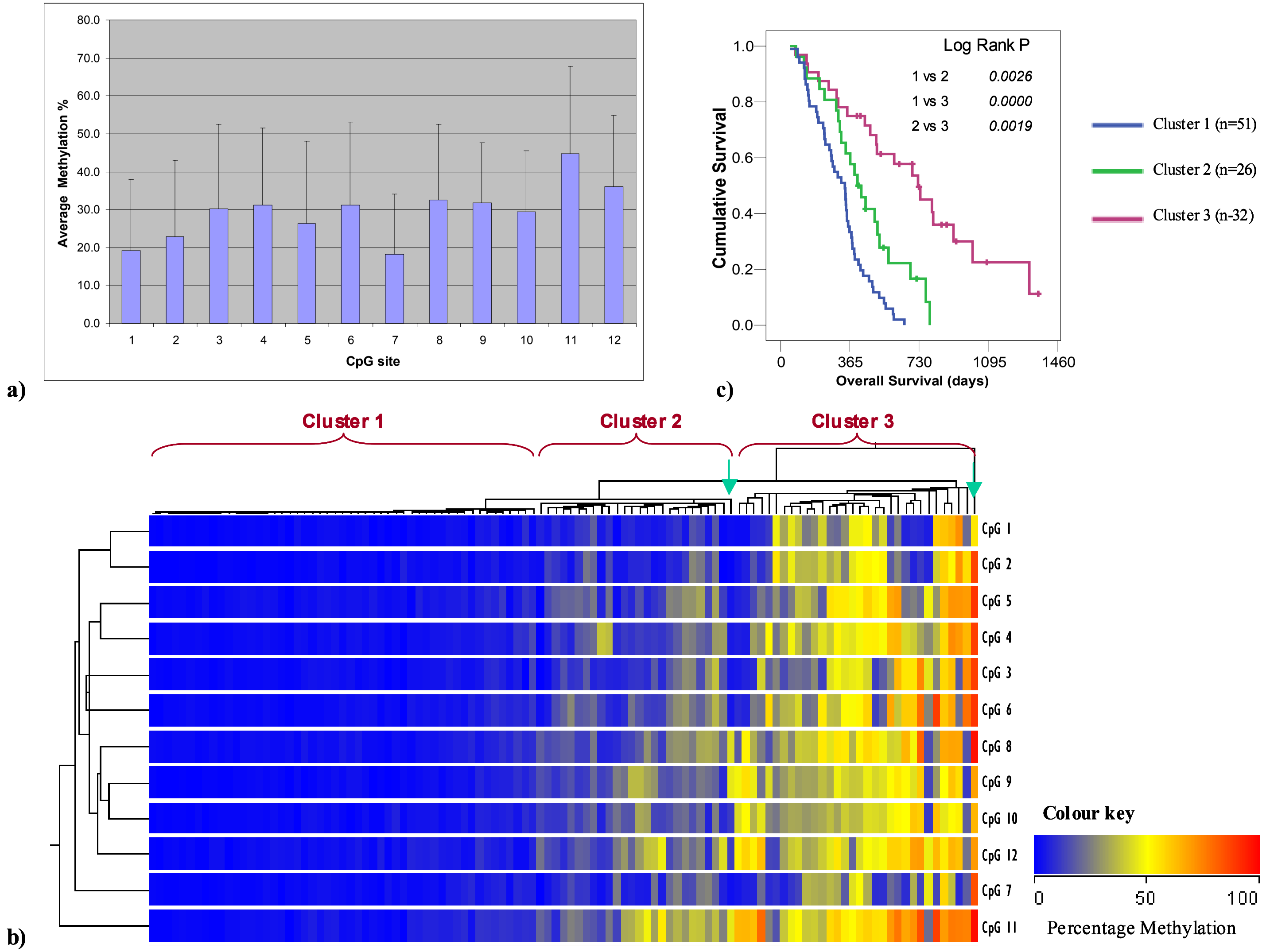


**Figure 4 a)** MGMT methylation at each CpG site shown as the average of all methylated tumour samples analysed ± standard deviation. **b)** Unsupervised hierarchical cluster analysis of average methylation at each CPG site for all cases in the clinical cohort treated with temozolomide and radiotherapy (performed in GeneSpring using euclidean distance and centroid distance) identified three main clusters. Cluster1 corresponded to tumours classed as unmethylated; cluster 2 had cases with lower levels of methylation; cluster 3 consisted of cases with higher levels of methylation seen in the majority of CpGs. Two cases (green arrows) clustered separately; these were included in cluster 2 or cluster 3 for comparisons with outcome data. **c)** Kaplan-Meier plot showing associations of methylation clusters with overall survival. Cases in cluster 3 had median survival 23.8 months (95% CI 17.3-30.4) and 2-year survival 49.5%. Cases in cluster 2 had median survival 13.3 months (95% CI 10.1-16.5) and 2-year survival 16.7%. Cases in cluster 1 had median survival 11.1 months (95% CI 8.8-13.3) and 2-year survival 0%. Progression-free survival was significantly different in clusters 1 vs 3 (log-rank P =0.0000) and 3 vs 2 (log-rank P=0.0000 ) (data not given).
